# Identification of inhibitors of an unconventional *Trypanosoma brucei* kinetochore kinase

**DOI:** 10.1371/journal.pone.0217828

**Published:** 2019-05-31

**Authors:** Leah S. Torrie, Fabio Zuccotto, David A. Robinson, David W. Gray, Ian H. Gilbert, Manu De Rycker

**Affiliations:** Drug Discovery Unit, Wellcome Centre for Anti-Infectives Research, School of Life Sciences, University of Dundee, Dundee, United Kingdom; Istituto di Genetica Molecolare, ITALY

## Abstract

The discovery of 20 unconventional kinetochore proteins in *Trypanosoma brucei* has opened a new and interesting area of evolutionary research to study a biological process previously thought to be highly conserved in all eukaryotes. In addition, the discovery of novel proteins involved in a critical cellular process provides an opportunity to exploit differences between kinetoplastid and human kinetochore proteins to develop therapeutics for diseases caused by kinetoplastid parasites. Consequently, we identified two of the unconventional kinetochore proteins as key targets (the highly related kinases KKT10 and KKT19). Recombinant *T*. *brucei* KKT19 (*Tb*KKT19) protein was produced, a peptide substrate phosphorylated by *Tb*KKT19 identified (KKLRRTLSVA), Michaelis constants for KKLRRTLSVA and ATP were determined (179 μM and 102 μM respectively) and a robust high-throughput compatible biochemical assay developed. This biochemical assay was validated pharmacologically with inhibition by staurosporine and hypothemycin (IC_50_ values of 288 nM and 65 nM respectively). Surprisingly, a subsequent high-throughput screen of a kinase-relevant compound library (6,624 compounds) yielded few hits (8 hits; final hit rate 0.12%). The low hit rate observed was unusual for a kinase target, particularly when screened against a compound library enriched with kinase hinge binding scaffolds. In an attempt to understand the low hit rate a *Tb*KKT19 homology model, based on human cdc2-like kinase 1 (CLK1), was generated. Analysis of the *Tb*KKT19 sequence and structure revealed no obvious features that could explain the low hit rates. Further work will therefore be necessary to explore this unique kinetochore kinase as well as to assess whether the few hits identified can be developed into tool molecules or new drugs.

## Introduction

Kinetochores are multiprotein complexes that associate with the centromere of a chromosome during cell division, ensuring faithful transmission of genetic material to daughter cells [1, 2]. While kinetochores are evolutionary quite plastic, most eukaryotic kinetochores share the same canonical kinetochore machinery [3]. The kinetochores of the protozoan parasite *Trypanosoma brucei*, the causative agent of human African trypanosomiasis [4], are an exception to this. The discovery of 20 novel kinetochore proteins in *T*. *brucei* [5–7], with no detectable homology to conventional eukaryotic kinetochore proteins, raises not only an interesting area of research to investigate the evolutionary history of these proteins, but also the possibility of exploiting these unique proteins in the search for new therapeutics, in particular as these proteins are conserved across the kinetoplastida order. This includes *Leishmania spp*. and *Trypanosoma cruzi*, the causative agents of leishmaniasis and Chagas disease [8, 9]. These neglected and devastating diseases affect some of the world’s poorest populations with current drug treatments suffering from high cost, host toxicity and emerging resistance [10, 11]. In the search for new therapeutics for these diseases, targeting novel, but essential, enzymes within the parasite provides an attractive start point for drug discovery. The finding that four of the kinetoplastid kinetochore proteins (KKTs) are kinases offers an opportunity to exploit a known, druggable enzyme family, whilst also targeting proteins unique to the parasite.

Of the four kinetochore kinases previously described [5, 6] (KKT2 (Tb927.11.10520), KKT3 (Tb927.9.10920), KKT10 (Tb927.11.12410) and KKT19 (Tb927.11.12420)), the KKT2 and KKT3 proteins were found to be similar in terms of domain organisation and amino acid sequence (48% similarity), as were the KKT10 and KKT19 proteins (also referred to as CLK1 and CLK2 respectively [12, 13]) (85% similarity), with these pairs most probably arising from gene duplication events. Genome-wide RNAi studies in *T*. *brucei* reveal that knockdown of these kinetochore kinases is associated with loss-of-fitness of the parasite [14, 15]. Further studies confirm that knockdown of protein levels with either KKT2, KKT10 or dual KKT10 / KKT19 RNAi constructs affect cell growth [5, 12, 13]. Interestingly, despite a genome-wide RNAi screen showing loss-of-fitness when KKT19 alone is knocked down [14, 15], subsequent KKT19 RNAi studies in *T*. *brucei* have revealed no effect on parasite growth [13, 16].

Chemical validation of the KKT10 protein has been achieved using the natural product hypothemycin [13]. This compound was shown to inhibit both *T*. *brucei* KKT10 (*Tb*KKT10 / *Tb*CLK1) activity (IC_50_ = 150 nM) and *T*. *brucei* cell growth (EC_50_ = 170 nM), with chemoproteomics confirming hypothemycin engages primarily with *Tb*KKT10 in *T*. *brucei* cell lysates at concentrations relevant to cellular efficacy [13]. Hypothemycin was also shown to reduce parasitemia in *T*. *brucei* infected mice, with prolonged survival of infected mice over 30 days and a 33% cure rate observed following 7 daily treatments with 10 mg/ml hypothemycin [13].

Based on these data, the KKT10 / KKT19 proteins were prioritised as targets for entry into a kinetoplastid drug discovery program. These kinases have been classified as members of the LAMMER subfamily of CMGC kinases [17] with 100% sequence identity in the active site. As such, any inhibitors identified would be expected to inhibit both kinases which, as highlighted in RNAi studies, confers a growth defect in *T*. *brucei* parasites [5]. Due to the successful production of recombinant *Tb*KKT19 protein, the current study focuses on the development of a high-throughput screening assay for *Tb*KKT19 and the subsequent high-throughput screen of a kinase-relevant compound library to identify ATP site inhibitors of this unique kinetoplastid kinetochore kinase.

## Materials and methods

### Protein expression and purification method for recombinant *Tb*KKT19

A His-tagged, full-length *Tb*KKT19 expression construct (kindly provided by Bungo Akiyoshi) was transformed into Rosetta 2 competent cells for protein production. A 60 ml overnight culture was set up and grown at 37°C 200 rpm for 16 h and was used the next day to inoculate 6 litres of LB+AMP+Kan media. Cells were grown at 37°C until the OD600 = 0.704 then induced with 0.2 mM ITPG then grown 20°C for 16 hrs before harvesting by centrifugation at 3,500 g for 30 min and storage at -20°C. Lysis buffer (60 ml, 50 mM NaPhosphate / 500 mM NaCl / 10% Glycerol / 20 mM Imidazole pH 7.5 / protease inhibitor tablets / DNAase) was added and the pellets defrosted at 25°C in a water bath for approximately 20 min. The slurry was then passed through a Cell Disrupter (Constant Systems) set at 30 KPSI to lyse the cells. The sample was then centrifuged at 40,000 g for 30 min. The supernatant was then filtered using syringe filters to 0.2 μm. The supernatant was loaded onto a 5 ml HiTrap Ni HP column that had been equilibrated with Buffer A (50 mM NaPhosphate / 500 mM NaCl / 10% Glycerol / 20 mM Imidazole pH 7.5) at 5 ml/min using an AKTA Pure (GE). Once loaded the column was washed with 10 column volumes of buffer A followed by 5% Buffer B (50 mM NaPhosphate / 500 mM NaCl / 10% Glycerol / 500 mM Imidazole pH 7.5) to wash off His-rich contaminating proteins. A linear gradient of 5–50% B was used to elute the protein. Approximately 38 mg of protein (from 6 L culture) was present in the fractions containing the *Tb*KKT19 protein. The sample was then passed through a 0.2 μm filter, before loading onto a XK26/60 Superdex 75 column using an AKTA Pure at Room Temp at 2 ml/min.

### *Tb*KKT19 substrate identification

Following production of recombinant protein, *Tb*KKT19 was screened against a panel of 41 putative kinase peptide substrates (at a concentration of 300 μM or 100 μM for peptide KTFCGTPEYLAPEVRREPRILSEEEQEMFRDFDYIADWC) and 3 putative protein substrates (at a concentration of 1 mg/ml) using the kinase screening service provided by the MRC PPU International Centre for Kinase Profiling at the University of Dundee (S1 Table).

### *Tb*KKT19 biochemical assay development

The peptide substrate KKLRRTLSVA was custom synthesised by Pepceuticals Ltd. and used for further investigation in a non-radiometric high-throughput compatible assay. Activity of the *Tb*KKT19 enzyme was determined by monitoring levels of ADP released during the enzymatic reaction using the commercially available ADP Hunter Plus kit (DiscoverX).

Development of the *Tb*KKT19 assay was carried out in black, low-volume, 384-well plates (Greiner) at room temperature (~23°C). Enzyme linearity was assessed in a 10 minute end-point assay with varying *Tb*KKT19 concentrations (0–20 nM), 200 μM ATP and 200 μM peptide substrate KKLRRTLSVA (‘KKL’ peptide).

Assays to determine the apparent Michaelis constants (*K*_m_^app^) for the ATP and the ‘KKL’ peptide were carried out in 8 μl reaction volumes containing 5 nM recombinant *Tb*KKT19 prepared in the proprietary ADP Hunter buffer (pH 7.4) supplemented with 1 mM dithiothreitol. To determine the *K*_m_^app^ of ATP, the ATP concentration was varied (0–800 μM), with the ‘KKL’ concentration fixed at a saturating concentration of 1 mM. To determine the *K*_m_^app^ of the ‘KKL’ peptide, the ‘KKL’ concentration was varied (0–800 μM), with the ATP concentration fixed at a saturating concentration of 600 μM. Detection of ADP formation was carried out by the addition of 4 μl ADP Hunter kit reagent A, followed by the addition of 8 μl ADP Hunter kit reagent B to each assay well. The ADP Hunter signal was allowed to develop for 30 min before the fluorescence intensity (FI) of each well was read using a PheraStar plate reader (BMG) (Excitation 540 nm; Emission 590 nm). Assays were monitored over time (0–60 min), with linear reaction rates at each substrate concentration determined. Rate *versus* substrate concentration data were fitted to the Michaelis-Menten equation using GraFit (Erithacus Software).

### *Tb*KKT19 hit identification

The *Tb*KKT19 high throughput screen was performed using our in-house kinase-relevant compound collection, containing 6,624 compounds [18]. All library compounds were solubilized in 100% DMSO to a concentration of 10 mM.

Single point inhibition assays were carried out at room temperature (~23 °C) in black, low-volume, 384-well plates (Greiner). Each assay was performed in an 8 μl reaction volume containing ADP Hunter buffer (pH 7.4), 1 mM dithiothreitol, 5 nM *Tb*KKT19, 80 μM ATP, 100 μM ‘KKL’ peptide, and 30 μM test compound.

Test compounds (24 nl in DMSO) were transferred to assay plates using an ECHO 550 acoustic dispenser (Labcyte). DMSO was added to 0% inhibition control wells, with staurosporine added to 100% inhibition control wells (to a final assay concentration of 30 μM). Assays were performed by adding 4 μl buffer with enzyme to all assay plates before the reaction was initiated with the addition of a 4 μl substrate mix containing ATP and ‘KKL’ peptide to all wells. Following a 30 min reaction at room temperature the assay was stopped with the addition of 4 μl ADP Hunter kit reagent A, followed by the addition of 8 μl ADP Hunter kit reagent B. The ADP Hunter signal was allowed to develop for 30 min before the FI of each well was read using a PheraStar plate reader (BMG) (Excitation 540 nm; Emission 590 nm). All liquid dispensing steps were carried out using a Thermo Scientific Multidrop dispenser (Matrix).

ActivityBase from IDBS was used for data processing and analysis. Test compound data were normalised to 0% inhibition and 100% inhibition control wells on each plate, with compounds designated as hits if the % inhibition at 30 μM was >35%.

To generate IC_50_ data for *Tb*KKT19 hit compounds, 10-point inhibitor curves were prepared in 384-well assay plates using an ECHO 550 acoustic dispenser (Labcyte). Following preparation of the inhibitor curves, assays were carried out using the ADP Hunter assay as described above.

ActivityBase from IDBS was again used for data processing and analysis. All IC_50_ curve fitting was undertaken using ActivityBase XE from IDBS. A four-parameter logistic dose-response curve fit was utilized with prefit used for all four parameters.

### *Tb*KKT19 counterscreen

As described above, 10-point inhibitor curves were prepared in 384-well plates using an ECHO 550 acoustic dispenser (Labcyte). Counterscreen assays, to identify any compounds inhibiting the ADP Hunter detection components rather than *Tb*KKT19, were carried out by adding 8 μl of 24 μM ADP, prepared in ADP Hunter buffer (pH 7.4) containing 1 mM dithiothreitol, to all wells, with the exception of the 100% effect control wells, to which 8 μl buffer only was added.

Following a 30 min incubation at room temperature (~23°C), detection of ADP levels using the ADP Hunter kit was carried out as previously described. All liquid dispensing steps were carried out using a Thermo Scientific Multidrop dispenser (Matrix).

Data processing and analysis was performed using ActivityBase from IDBS as described above.

### Kinase-related screening set

The screening set contains 6,624 compounds containing a pharmacophore that could interact with the hinge in the ATP binding site based on principles laid out in [18]. For the compounds in the screening library a set of molecular properties comprising molecular weight (MW), hydrogen bond acceptors and donors count (HBA, HBD), rotatable bonds count (Rb), aromatic rings count (Ar), number of heavy atoms and AlogP were calculated using BIOVIA Pipeline Pilot. To evaluate the scaffold distribution, compounds were clustered within Pipeline Pilot using their Bemis-Murcko assembly representation [19]. The clustering was carried out using FCFP4 as molecular descriptors and Tanimoto similarity 0.4 as maxim distance from cluster seed.

### *Tb*KKT19 modelling

#### Homology model

The *Tb*KKT19 sequence was downloaded from the NCBI database (accession code: XP_829304.1—see SI) and was used to query the PDB using “BLAST” as implemented in the NCBI blastp suite (https://blast.ncbi.nlm.nih.gov/) to identify suitable template structures to build the *Tb*KKT19 model. The structure of the human cdc2-like kinase 1 (*h*CLK1) was selected as template. An alignment between the target sequence *Tb*KKT19 and the sequence extracted from the *h*CLK1 structure was generated using Schrödinger Prime and manually curated. The optimised sequence alignment was used to build the homology model using the knowledge-based method in Prime. Model refinement was performed by Molecular Dynamics using Schrödinger Desmond.

#### Hit compounds docking

The three-dimensional structures of the hits were built in Schrödinger Maestro, minimized with the OPLS3 force field [20] and docked in the catalytic site of the *Tb*KKT19 model using Schrödinger GLIDE-SP.

## Results

### Enzymatic characterisation of *Tb*KKT19

**Fig 1:**
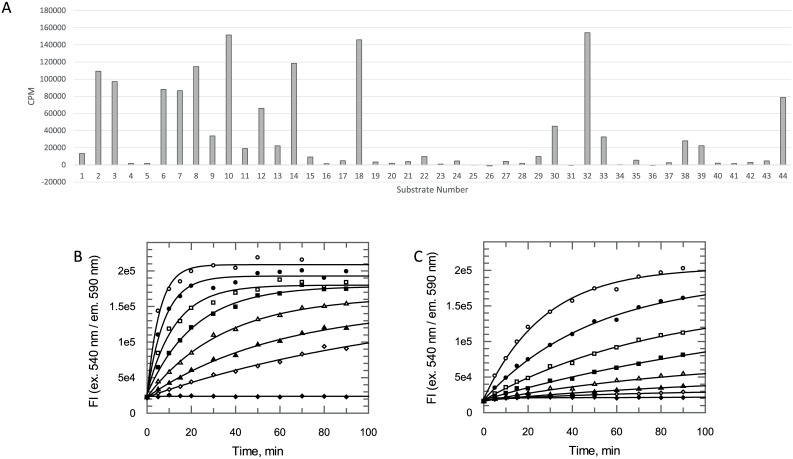
*Tb*KKT19 substrate search. (A) A panel of 41 peptides and 3 proteins (see S1 Table for details) were screened as possible substrates of *Tb*KKT19 in a radiometric kinase assay performed by the MRC PPU International Centre for Kinase Profiling at the University of Dundee (data shown as mean counts per minute above background (CPM) of two technical replicates). (B) Confirmation of peptide substrate activity using ADP Hunter Plus assay kit (DiscoverX). The activity of various *Tb*KKT19 concentrations monitored over time using 200 μM KKLRRTLSVA (peptide 8 in panel A) and 200 μM ATP (data points are *n* = 1). (C) Activity of various *Tb*KKT19 concentrations monitored over time in the presence of 200 μM ATP revealed ADP formation can be detected over time in the absence of ‘KKL’ peptide substrate (data points are *n* = 1). In panels (B) and (C) *Tb*KKT19 concentrations are 175 nM (open circles), 88 nM (closed circles), 44 nM (open squares), 22 nM (closed squares), 11 nM (open triangles), 5.5 nM (closed triangles), 2.7 nM (open diamonds) and 0 nM (closed diamonds).

Recombinant, His-tagged, full-length *Tb*KKT19 protein was produced (S1 Fig) and a panel of putative kinase substrates tested to identify a suitable peptide phosphorylated by *Tb*KKT19 (Fig 1A and S1 Table). On the basis of its availability in the laboratory, KKLRRTLSVA (‘KKL’ peptide) was selected for further investigation. Using an assay that measures ADP production, ‘KKL’ peptide was confirmed to be phosphorylated by *Tb*KKT19 (Fig 1B) and was used as a peptide substrate in biochemical *Tb*KKT19 assays. In addition, it was also noted that ADP formation could be detected in the absence of the ‘KKL’ peptide (Fig 1C), which may indicate autophosphorylation of the enzyme. Alternatively *Tb*KKT19 may display intrinsic ATPase activity in the absence of peptide substrate, a feature previously observed in other CMGC kinases [21, 22]. For future assay development a concentration of 5 nM *Tb*KKT19 was selected, as ADP formation in the absence of ‘KKL’ peptide could not be detected using this enzyme concentration, whereas phosphorylation of the ‘KKL’ peptide gave a robust and measurable assay signal.

**Fig 2:**
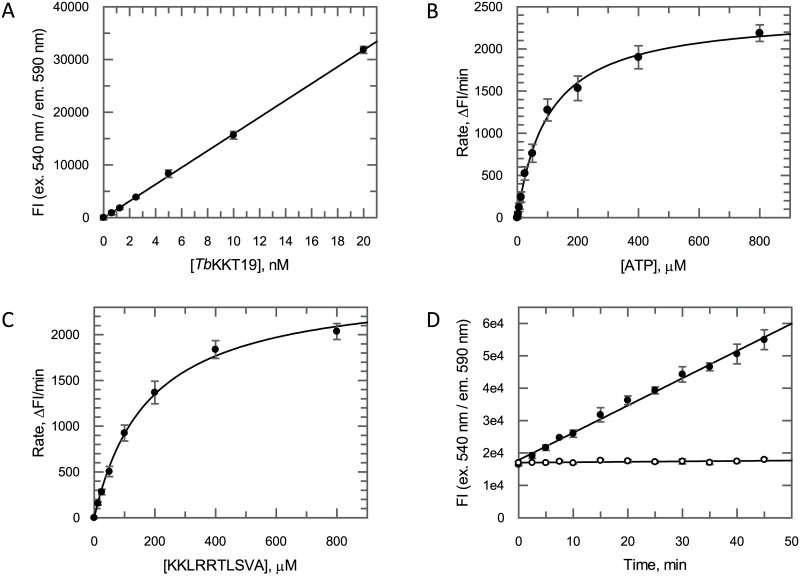
*Tb*KKT19 assay development summary. (A) Linearity of the *Tb*KKT19 assay with respect to enzyme concentration. Data are shown as mean FI ± SD (*n* = 3 technical replicates). (B) ATP *K*_m_^app^ determination in the presence of a saturating concentration of 1 mM ‘KKL’ peptide. Data are shown as rate ± SE of linear fit (rates determined from *n* = 2 technical replicates). (C) ‘KKL’ peptide *K*_m_^app^ determination in the presence of a saturating concentration of 600 μM ATP. Data are shown as rate ± SE of linear fit (rates determined from *n* = 2 technical replicates). (D) Assay linearity with respect to time under the final assay screening conditions of 5 nM *Tb*KKT19, 100 μM ‘KKL’ peptide and 80 μM ATP either with (open circles) or without (closed circles) 30 μM staurosporine. Data are shown as mean FI ± SD (*n* = 3 technical replicates).

**Fig 3:**
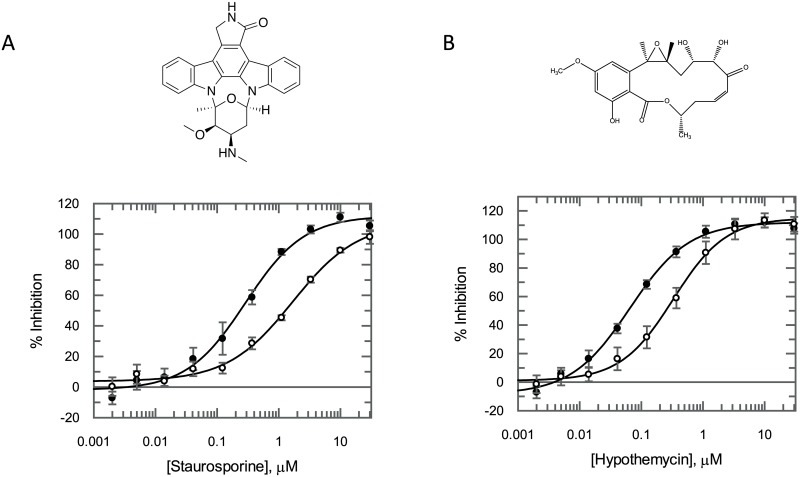
Staurosporine and hypothemycin inhibit *Tb*KKT19. (A) Staurosporine inhibits *Tb*KKT19 with an IC_50_ of 288 nM (95% CI of best fit 190–386 nM) in the presence of 80 μM ATP (closed circles) and 1.68 μM (95% CI of best fit 0.99–2.37 μM) in the presence of 800 μM ATP (open circles). (B) Hypothemycin inhibits the *Tb*KKT19 enzyme with an IC_50_ of 65 nM (95% CI of best fit 50–80 nM) in the presence of 80 μM ATP (closed circles) and 328 nM (95% CI of best fit 267–389 nM) in the presence of 800 μM ATP (open circles). The 5.8-fold and 5.0-fold shifts in potency for staurosporine and hypothemycin respectively with increasing ATP concentrations are consistent with ATP competitive inhibition. All data points presented as mean % inhibition ± SD (*n* = 3 technical replicates).

Using the ‘KKL’ peptide as a substrate, the *Tb*KKT19 enzyme was characterised and assay conditions optimised to ensure appropriate inhibitor screening conditions were used. This involved determining the optimal enzyme concentration, assessing assay linearity with respect to time, and determining apparent Michaelis constants (*K*_m_^app^) for the substrates (Fig 2). The ATP and ‘KKL’ peptide *K*_m_^app^ values were determined to be 102 μM (95% CI 87–117 μM) and 179 μM (95% CI 150–208 μM) respectively. Pharmacological validation of the assay was achieved using both the pan-kinase inhibitor staurosporine and the *Tb*KKT10 (*Tb*CLK1) inhibitor hypothemycin [13]. Staurosporine and hypothemycin inhibited *Tb*KKT19 with IC_50_ values of 288 nM (95% CI 190–386 nM) and 65 nM (95% CI 50–80 nM) respectively (Fig 3, closed circles). In addition, when the ATP concentration was increased 10-fold from 80 μM to 800 μM, 5 to 6-fold shifts in these IC_50_ values were observed (1.68 μM (95% CI 0.99–2.37 μM) and 328 nM (95% CI 267–389 nM) for staurosporine and hypothemycin respectively), indicative of ATP competitive binding (Fig 3, open circles).

### *Tb*KKT19 hit discovery

For high-throughput screening a non-biased approach was taken, with screening concentrations of 80 μM ATP and 100 μM ‘KKL’ peptide selected (*i*.*e*. substrate concentrations slightly below their respective *K*_m_^app^, allowing inhibitors with any inhibition modality to be identified). The screen was carried out with a kinase-relevant library of 6,624 compounds, characterised by the presence of a pharmacophore that could interact with the hinge motif in the ATP binding site. All compounds are Lipinski rule-of-five compliant [23] and Table 1 shows the mean values of the calculated molecular parameters; the mean values are well below the Lipinski values, allowing for further development. The library is chemically diverse and includes 957 clusters and 969 singletons. The largest cluster contains 191 analogues whilst on average there are 6 molecules in each cluster. Examples of the most populated scaffolds are reported in S2 Fig. The library was screened in single replicate at a concentration of 30 μM. To assess the robustness and reproducibility of the assay and the quality of the hit discovery campaign, various criteria were assessed following completion of the primary screen. These data reveal a high quality screening campaign, with a mean *Z*′ [24] of 0.88 ± 0.03 and a mean signal to background ratio of 2.69 ± 0.12.

**Table 1:** Calculated properties for kinase-relevant compound library.

Molecular property	Mean value
Molecular weight	320.35
HBA count	4.1
HBD count	1.2
Heavy atoms count	23
Rotatable bonds count	3.9
Number of aromatic rings	2.7
AlogP	2.67

**Fig 4:**
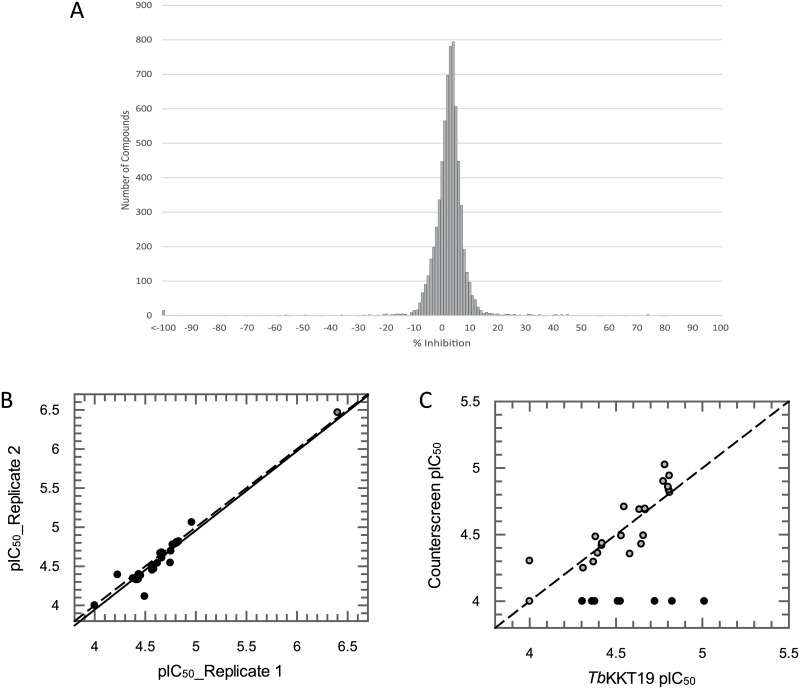
*Tb*KKT19 hit discovery summary. (A) Frequency distribution of compound percentage inhibition values. An arbitrary hit identification cut-off of 35% inhibition identified 31 putative hit molecules out of 6,624 molecules screened (0.46% hit rate). (B) Correlation between two independent pIC_50_ (−log IC_50_ (M)) determinations for each of the 31 hit compounds. Black circles represent test compounds, grey circle is staurosporine control. Dashed line is line of equipotence; solid line is linear regression of these data with a correlation coefficient of 0.93. (C) Correlation between mean *Tb*KKT19 pIC_50_ from two independent determinations and the pIC_50_ measured in an ADP Hunter technology interference counterscreen. Black circles represent true *Tb*KKT19 hits (inactive in counterscreen assay), grey circles represent compounds displaying technology interference. Dashed line is line of equipotence.

Hit compounds were identified by applying an arbitrary threshold cut-off of 35% inhibition with 31 compounds from the kinase library meeting this criteria (0.46% hit rate) (Fig 4A). Hit compounds were cherry picked and retested as 10-point dose response curves in the *Tb*KKT19 assay to determine IC_50_ values (potencies in the range of 9.8–100 μM determined) (Fig 4B). In addition, hit compounds were also tested in a counterscreen assay to exclude compounds that inhibit the ADP Hunter assay technology. Following this assessment only 8 confirmed kinase library hits remained (Fig 4C), corresponding to a final hit rate of 0.12%.

**Fig 5:**
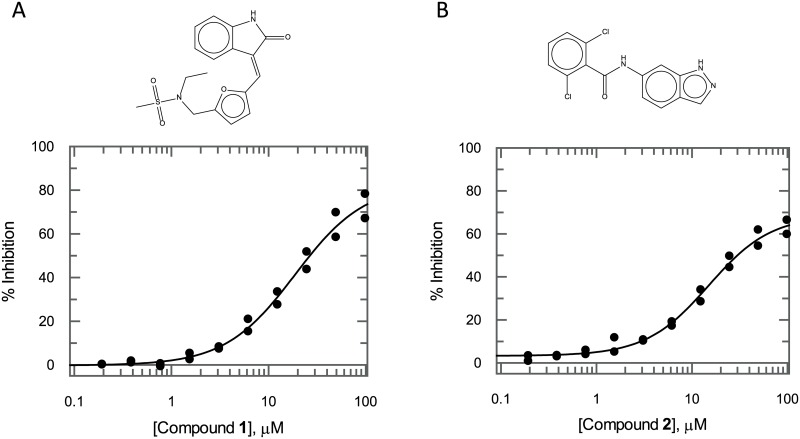
Compound 1 and compound 2 inhibit *Tb*KKT19. (A) Compound **1** and (B) compound **2** inhibit *Tb*KKT19 with IC_50_ values of 18.9 μM (95% CI of best fit 16.5–21.6 μM) and 14.9 μM (95% CI of best fit 14.5–15.4 μM) respectively when screened using final assay conditions of 5 nM *Tb*KKT19, 100 μM ‘KKL’ peptide and 80 μM ATP. All data points shown (*n* = 2 biological replicates).

Two of the most potent hits (compound **1** and compound **2**; Fig 5) returned IC_50_ values of 18.9 μM (95% CI 16.5–21.6 μM) and 14.9 μM (95% CI 14.5–15.4 μM) respectively. Both compounds were repurchased and reconfirmed as hits in the *Tb*KKT19 biochemical assay, however, due to their weak potency against the *Tb*KKT19 enzyme, no meaningful *T*. *brucei* cell-based data could be generated for these compounds.

### *Tb*KKT19 homology model

To understand the binding mode of our hit compounds and to try to account for the low hit rate observed for this kinetochore kinase, a *Tb*KKT19 homology model was generated. The *Tb*KKT19 sequence used to identify suitable 3D template structures using NCBI BLAST is shown in S3 Fig. Several human cdc2-like kinase (CLK) structures (e.g. *h*CLK3—PDB codes 2eu9; 2exe, 2wu6, 2wu7h—sequence identity 34%; *h*CLK1—PDB code: 1z57—sequence identity 34%; *h*CLK2—PDB code 3nr9—sequence identity 34%) were identified as suitable template structures for homology modelling. After inspection of the highly homologous structures only structures containing small molecule ligands were considered. Apo structures (e.g. PDB code 2eu9), structure with missing N lobe (PDB code 2exe) or structures with compounds not matching the hinge pharmacophore [25] (e.g. PDB code 2vag) were excluded. Finally, the structure of the human cdc2-like kinase 1 (*h*CLK1) complexed with 10Z-Hymenialdisine [PDB code: 1z57] [26] was selected as a template to build the *Tb*KKT19 model.

**Fig 6:**
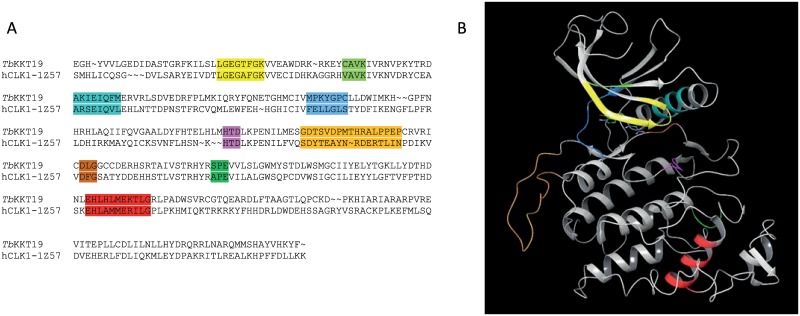
Sequence alignment and *Tb*KKT19 homology model. In yellow, the xGxGxxGx motif of the Gly-rich loop, in light green the xAVK motif of the highly conserved catalytic Lys, in teal the α-C helix with the conserved catalytic Glu, in blue the hinge region starting with the gatekeeper residue, in magenta the HxD motif, in brown the DxG motif and in dark green the xPE motif. In orange the β-hairpin insertion and in red the LAMMER motif typical of the cdc2-like kinase family.

An alignment between the target sequence *Tb*KKT19 and the sequence extracted from the *h*CLK1 structure (Fig 6A) was generated. Despite a relatively low level of sequence conservation, kinases are characterised by a high degree of structural similarity. The alignment was manually curated to remove gaps / insertions from secondary structural elements and to ensure that the typical kinase sequence motifs important for catalysis and regulation were correctly aligned. A homology model for *Tb*KKT19 was then generated based on *h*CLK1 structure using the optimised sequence alignment. The homology model was further optimised by energy minimisation and further validated by molecular dynamics (MD). An MD simulation of 100 ns showed that the system is well equilibrated and the template ligand is stably bound to the ATP pocket interacting with the hinge and the conserved catalytic Lys and Asp of the DLG motif (S4 Fig).

### Compound 1 and compound 2 binding mode

Both validated hit compounds (Fig 5) contain well-characterised hinge-binder chemical scaffolds. For instance, compound **2** contains an indazole ring that is also present in the kinase inhibitor entrectinib, currently in Phase II against NTRK/ROS1/ALK driven tumours, whereas the *trans*-3-ethylideneindolin-2-one group in compound **1** is also the hinge binding moiety in sunitinib, an inhibitor of receptor tyrosine kinases, currently on the market for the treatment of renal cell carcinoma and gastrointestinal stromal tumours.

**Fig 7:**
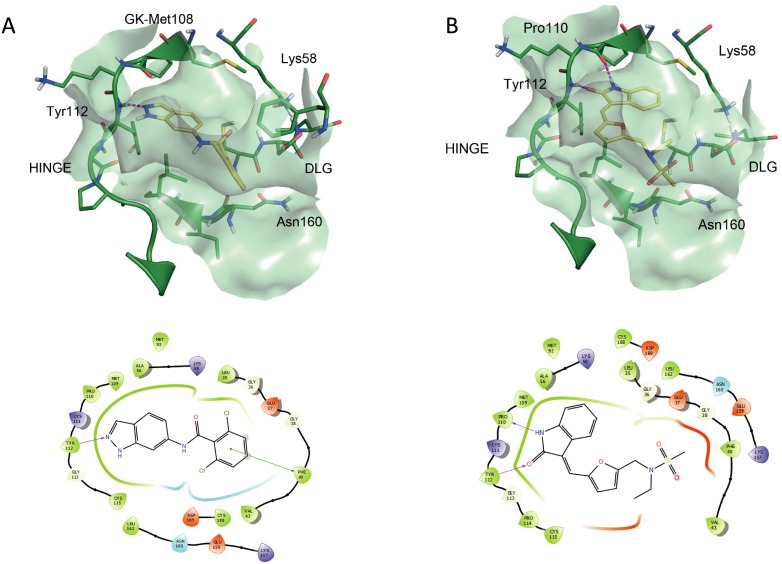
Docking study results. (A) Binding mode and 2D interaction diagram for compound **1** and (B) binding mode and 2D interaction diagram for compound **2**. Ligand is characterised by yellow carbon atoms. Red arrows/dashed lines indicate hydrogen bonding interactions, green line indicate π-stacking.

The binding mode for both compounds in *Tb*KKT19 was investigated. The docking model for compound **1** (Fig 7A) is consistent with the mode of binding of sunitinib in different kinases (e.g. KIT, CDK2). The indolinone scaffold establishes two hydrogen bond interactions with the hinge motif of KKT19. One between the Tyr112 backbone NH and the carbonyl oxygen of the ligand and one between the Pro110 backbone carbonyl oxygen and the lactam NH. The phenyl ring of the indolinone moiety faces the gatekeeper residue Met 109. The double bond in compound **1** is trans whereas the equivalent bond in sunitinib is cis. That results in compound **1** developing into the ATP binding site following a different vector and placing the furan ring in the sugar subpocket. The indazole scaffold of compound **2** also occupies the adenine pocket of the ATP binding site and interacts with the Tyr112 backbone NH in the hinge (Fig 7B). The di-o-Cl-phenyl moiety is positioned underneath the Gly-rich loop establishing π-stacking with Phe40.

### Analysis of low hit rate

As the low hit-rate in the kinase-relevant compound library screen was unexpected we looked for possible explanations. An analysis of the *Tb*KKT19 sequence highlighted a number of unusual features (Fig 6). The beginning of the kinase activation loop is normally characterised by the DFG motif. The aspartate residue is crucial for the binding of divalent cations that coordinate the ATP phosphate groups whereas the phenylalanine of the DFG motif plays an important role in regulatory mechanisms and catalytic efficiency as it is part of the regulatory hydrophobic spine (R-spine), a highly conserved spatial motif comprising four non-consecutive hydrophobic residues [27]. The structural assembly of the R-spine is a requirement for activated kinases. Hence, formation of the R-spine is highly regulated and typically requires phosphorylation of the activation loop. Together with the catalytic hydrophobic spine (C-spine) and the gatekeeper residue, the R-spine is functionally relevant [28]. In *Tb*KKT19 the activation loop starts with a DLG motif where the phenylalanine residue is replaced by a leucine. We hypothesised that the change in the hydrophobic spine amino-acid composition could have an impact on ATP *K*_m_. Kinases characterised by a low ATP *K*_m_ value can be difficult to target as small molecule compounds will have to be very potent to be able to compete with cellular ATP. However, we determined that *Tb*KKT19 has a relatively high ATP *K*_m_^app^ (102 μM), indicating that it should be possible to identify compounds capable of competing for the ATP binding site. The end of the *Tb*KKT19 activation loop is characterised by an SPE motif instead of the more common APE motif. The APE motif is important for catalysis as it interacts with the αF-helix stabilising the activation loop. The replacement of the alanine or proline residues of the APE motif is however not predicted to disrupt this interaction.

The *Tb*KKT19 sequence also presents a HTD motif instead of the common HRD motif. The HRD motif is a highly conserved motif in the catalytic loop, it is a key regulatory and substrate binding element as the histidine residue is also part of the hydrophobic spine and the aspartate is involved in the phospho transfer step [29]. The HRD replacement by a HTD motif is common in all members of the CLK family and does not seem to have an impact on the catalytic efficiency of the kinase [26].

The *Tb*KKT19 hinge region comprises the motif MPKYGPC. The presence of the two proline residues in position 1 and 5 relative to the gatekeeper residue Met109 (GK+1 and GK+5) was investigated to verify the impact on the hinge pharmacophore. The presence of Pro residues can alter the recognition pattern between the hinge and the ligand as the unique Pro geometry changes the orientation of the backbone features interacting with the ligand. A typical example is PIM1 where the presence of a proline in position GK+3 removes the key hydrogen bond acceptor feature from the hinge resulting in an unconventional kinase hinge pharmacophore. A 100 ns dynamic simulation was carried out showing that the proline residues in the *Tb*KKT19 hinge do not alter the recognition motif at the hinge. This is also consistent with the hinge geometry observed in other kinase structures with one Pro residues either in position GK+1 (e.g. MET) or position GK+5 (e.g. CK1).

Finally, an analysis of the *Tb*KKT19 sequence in relation to the *h*CLK1 structure used as template shows that the *Tb*KKT19 sequence matches a number of long insertions typical of the CLK family. In particular, residues 165–183 (Fig 6) are mapping to an extended β-hairpin at the top of the C-terminal lobe in the template structure. Residues 251–283 map onto a long insertion at the bottom of the C-terminal domain. In the CLK1 structure this insertion renders the αG-helix that includes part of the LAMMER motif that characterises the CLK family, solvent inaccessible. The LAMMER motif seems to be only partially conserved in *Tb*KKT19 (the EHLAMMERILG in human is EHLHLMEKTLG in *T*. *brucei*). These elements are important in the recognition of substrate but should not interfere with the binding of small molecules at the ATP site. Altogether, none of the *Tb*KKT19 specific sequence features appear to explain the unexpectedly low hit-rate.

## Discussion

KKT19 was previously identified as an unconventional kinetochore kinase in *T*. *brucei* [5]. Kinetic characterisation of this protein revealed it was enzymatically active as a kinase, with a substrate specificity profile similar to other reported CLK kinases (consensus sequence R-X-X-S) [26, 30, 31]. In addition, *Tb*KKT19 was inhibited by the pan-kinase inhibitor staurosporine as well as hypothemycin, a natural product known to inhibit kinases with a cysteine residue before the DXG motif [13], as seen for *Tb*KKT19. Hypothemycin was previously shown to inhibit *Tb*KKT10 and the IC_50_ generated here for *Tb*KKT19, in presence of 80 μM ATP, as well as the ATP-competitive profile, are highly comparable to the data published for *Tb*KKT10 (IC_50_ = 150 nM when assayed using 100 μM ATP [13]). This is not unexpected as both enzymes are closely related, and may well have redundant functions, it is therefore reassuring from a drug discovery perspective that dual inhibition of *Tb*KKT10 and *Tb*KKT19 is possible.

Despite appearing to function as a typical protein kinase and showing a pharmacological profile consistent with other kinases of the same family, a high-throughput screen of a kinase-relevant library yielded a very low hit rate. This result was unexpected as this library is composed of compounds with scaffolds designed to bind the ATP site of kinases and previous screens using a similar compound set with other kinases yielded higher hit rates (for example, the *T*. *brucei* GSK3 and *Leishmania* CRK3 kinases returned hit rates of 12.8% and 2.2% respectively) [32, 33]. The small number of *Tb*KKT19 hit molecules that we did identify contained known kinase hinge-binding motifs.

In the absence of a crystal structure, and to investigate the unexpectedly low hit rate further, a homology model of *Tb*KKT19 was generated, and docking of two key hits into the model was consistent with other known hinge-binding compounds. *Tb*KKT19 has a series of unusual amino acid sequences in conserved kinase motifs, but based on the homology model none of these should affect compound binding in the ATP-binding pocket. In conclusion, homology modelling of this kinetoplastid kinetochore kinase could not provide any explanation for the low hit rates observed and the divergent pharmacology between *Tb*KKT19 and other kinases should be investigated further.

The few hit molecules that we identified had low potency against the enzyme. In order to develop tool molecules for target validation or potential new drug candidates for HAT a significant amount of work will be required not only to increase potency but also to generate compounds with optimal solubility, metabolic stability, oral bio-availability and CNS penetration. While the homology model will be useful to guide such efforts ideally a crystal structure is generated to facilitate this.

## Supporting information

S1 Table Substrate search peptide sequences and proteins. (DOCX)

S1 Fig *Tb*KKT19 protein production. SDS-PAGE gel showing production of 91.2% pure His-tagged, full-length *Tb*KKT19 protein.
(DOCX)

S2 Fig Scaffold representation in kinase-relevant compound library. Compound structures exemplifying the chemical scaffolds in the 10 largest of the 957 clusters obtained from the 6,624 compounds in the kinase-relevant library.
(DOCX)

S3 Fig *T*. *brucei* KKT19 sequence. The *Tb*KKT19 sequence is shown (https://www.ncbi.nlm.nih.gov/protein/XP_829304.1). In green are the residues that have been incorporated in the homology model.
(DOCX)

S4 Fig *Tb*KKT19 molecular dynamics simulation. Interaction that the 10Z-Hymenialdisine compounds from the *h*CLK1 template structure establishes in the *Tb*KKT19 model. The percentage values indicate the proportion of time a specific interaction is present during an MD simulation of 100 ns.
(DOCX)
