# Supplementary material for: Identification of inhibitors of an unconventional Trypanosoma brucei kinetochore kinase
Source: PLoS One. 2019 May 31;14(5):e0217828. doi: 10.1371/journal.pone.0217828 (PMC6544269; doi:10.1371/journal.pone.0217828)
Supplement: S1 Table — (DOCX) [file pone.0217828.s001.docx]

**S1 Table. Substrate search peptide sequences and proteins**

| **Substrate Number** | **Peptide Sequence / Protein** | **Screening Concentration** |
| --- | --- | --- |
| **1** | KKLNRTLSFAEPG | 300 μM |
| **2** | KEAKEKRQEQIAKRRRLSSLRASTSKSGGSQK | 300 μM |
| **3** | RFRRSRRMI | 300 μM |
| **4** | ISDELMDATFADQEAKKK | 300 μM |
| **5** | RRKDLHDDEEDEAMSITA | 300 μM |
| **6** | KKLNRTLSVA | 300 μM |
| **7** | KRKQISVRGL | 300 μM |
| **8** | KKLRRTLSVA | 300 μM |
| **9** | [LRRLSLG]x4 | 300 μM |
| **10** | AALVRQMSVAFFFKKKKK | 300 μM |
| **11** | KKISGRLSPIMTEQ | 300 μM |
| **12** | KKRPQRATSNVFA | 300 μM |
| **13** | LRRASLG | 300 μM |
| **14** | RSRSRSRSRSRSRSR | 300 μM |
| **15** | RRRLSFAEPG | 300 μM |
| **16** | RKKFGESEKTKTKEFL | 300 μM |
| **17** | GRPRTSSFAEGKK | 300 μM |
| **18** | ERMRPRKRQGSVRRRV | 300 μM |
| **19** | HMRSAMSGLHLVKRR | 300 μM |
| **20** | KKKKERLLDDRHDSGLDSMKDEE | 300 μM |
| **21** | YLRRRLSDSNF | 300 μM |
| **22** | LSNLYHQGKFLQTFCGSPLYRRR | 300 μM |
| **23** | LDDRHDSGLDSMKDEEY | 300 μM |
| **24** | KKSRGDYMTMQIG | 300 μM |
| **25** | KVEKIGEGTYGVVYK | 300 μM |
| **26** | POLY (Glu Tyr 4:1) | 1 mg/ml |
| **27** | DGEFLRTSCGSPNYAARRR | 300 μM |
| **28** | RRRDDDSDDD | 300 μM |
| **29** | FLAKSFGSPNRAYKK | 300 μM |
| **30** | RNRYRDVSPFDHSR | 300 μM |
| **31** | EAIYAAPFAKKK | 300 μM |
| **32** | Myelin Basic Protein | 1 mg/ml |
| **33** | YRRAAVPPSPSLSRHSSPHQS*EDEEE | 300 μM |
| **34** | KKRNRTLTV | 300 μM |
| **35** | [YSPTSPS]X3KKK | 300 μM |
| **36** | RLGRDKYKTLRQIRQ | 300 μM |
| **37** | KTFCGTPEYLAPEVRREPRILSEEEQEMFRDFDYIADWC | 100 μM |
| **38** | RSRHSSYPAGT | 300 μM |
| **39** | Histone H1 | 1 mg/ml |
| **40** | KKKSPGEYVNIEFG | 300 μM |
| **41** | ALNRTSSDSALHRRR | 300 μM |
| **42** | RRHYYYDTHTNTYYLRTFGHNTRR | 300 μM |
| **43** | KKKKEEIYFFFG | 300 μM |
| **44** | KKKVSRSGLYRSPSMPENLNRPR | 300 μM |
