# Supplementary material for: Identification of inhibitors of an unconventional Trypanosoma brucei kinetochore kinase
Source: PLoS One. 2019 May 31;14(5):e0217828. doi: 10.1371/journal.pone.0217828 (PMC6544269; doi:10.1371/journal.pone.0217828)
Supplement: S1 Fig — SDS-PAGE gel showing production of 91.2% pure His-tagged, full-length TbKKT19 protein. (DOCX) [file pone.0217828.s002.docx]

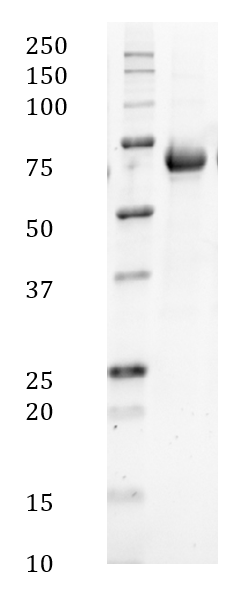


**S1 Fig.** ***Tb*KKT19 protein production.** SDS-PAGE gel showing production of 91.2% pure His-tagged, full-length *Tb*KKT19 protein.
