# Supplementary material for: Identification of inhibitors of an unconventional Trypanosoma brucei kinetochore kinase
Source: PLoS One. 2019 May 31;14(5):e0217828. doi: 10.1371/journal.pone.0217828 (PMC6544269; doi:10.1371/journal.pone.0217828)
Supplement: S2 Fig — Compound structures exemplifying the chemical scaffolds in the 10 largest of the 957 clusters obtained from the 6,624 compounds in the kinase-relevant library. (DOCX) [file pone.0217828.s003.docx]

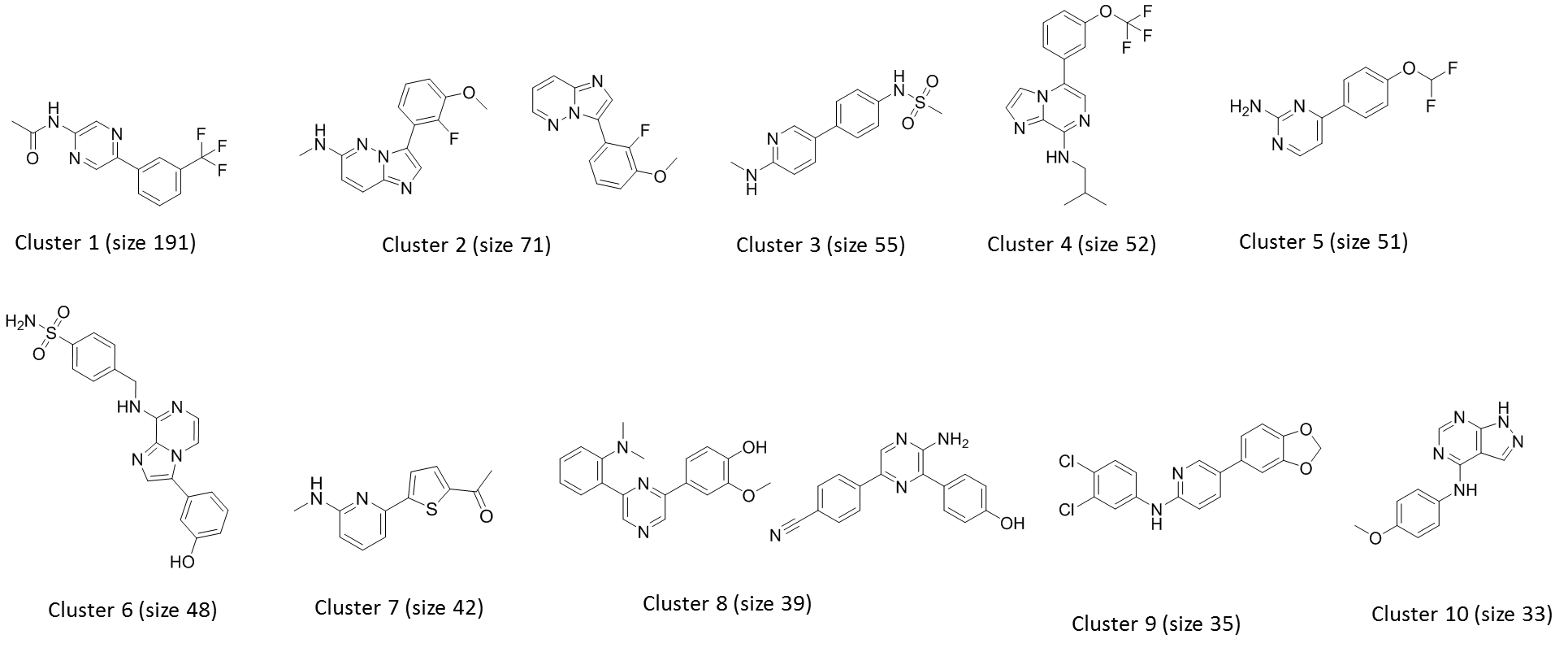


**S2 Fig.** **Scaffold representation in kinase-relevant compound library.** Compound structures exemplifying the chemical scaffolds in the 10 largest of the 957 clusters obtained from the 6,624 compounds in the kinase-relevant library.
