# Supplementary material for: Identification of inhibitors of an unconventional Trypanosoma brucei kinetochore kinase
Source: PLoS One. 2019 May 31;14(5):e0217828. doi: 10.1371/journal.pone.0217828 (PMC6544269; doi:10.1371/journal.pone.0217828)
Supplement: S3 Fig — The TbKKT19 sequence is shown (https://www.ncbi.nlm.nih.gov/protein/XP_829304.1). In green are the residues that have been incorporated in the homology model. (DOCX) [file pone.0217828.s004.docx]

>XP_829304.1 protein kinase, putative [Trypanosoma brucei brucei TREU927]

MASYIASNVNSSTIAKGMSGDTAGFYGSSTTAMVPPPVVIASKTNHPKNQQYQSTFQADERGITHPRRSA

SKRDRDNGIEQPSAIDQSATAANNSADSAVTPKSAHGVTSYASNKKKKVTYALPNQSREEGHFYVVLGED

IDASTGRFKILSLLGEGTFGKVVEAWDRKRKEYCAVKIVRNVPKYTRDAKIEIQFMERVRLSDVEDRFPL

MKIQRYFQNETGHMCIVMPKYGPCLLDWIMKHGPFNHRHLAQIIFQVGAALDYFHTELHLMHTDLKPENI

LMESGDTSVDPMTHRALPPEPCRVRICDLGGCCDERHSRTAIVSTRHYRSPEVVLSLGWMYSTDLWSMGC

IIYELYTGKLLYDTHDNLEHLHLMEKTLGRLPADWSVRCGTQEARDLFTAAGTLQPCKDPKHIARIARAR

PVREVITEPLLCDLILNLLHYDRQRRLNARQMMSHAYVHKYFPECRQHPNHVDNRSKLPPTPVM

**S3 Fig.** ***T. brucei* KKT19 sequence.** The *Tb*KKT19 sequence is shown (https://www.ncbi.nlm.nih.gov/protein/XP_829304.1). In green are the residues that have been incorporated in the homology model.
