# Supplementary material for: Identification of inhibitors of an unconventional Trypanosoma brucei kinetochore kinase
Source: PLoS One. 2019 May 31;14(5):e0217828. doi: 10.1371/journal.pone.0217828 (PMC6544269; doi:10.1371/journal.pone.0217828)
Supplement: S4 Fig — Interaction that the 10Z-Hymenialdisine compounds from the hCLK1 template structure establishes in the TbKKT19 model. The percentage values indicate the proportion of time a specific interaction is present during an MD simulation of 100 ns. (DOCX) [file pone.0217828.s005.docx]

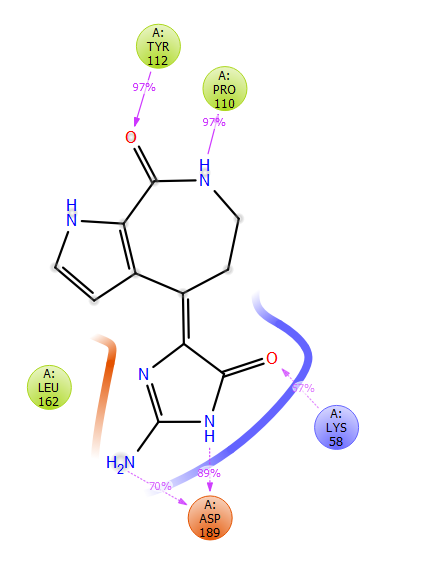


**S4 Fig.** ***Tb*KKT19 molecular dynamics simulation**. Interaction that the 10Z-Hymenialdisine compounds from the *h*CLK1 template structure establishes in the *Tb*KKT19 model. The percentage values indicate the proportion of time a specific interaction is present during an MD simulation of 100 ns.
